# Punctate Hippocampal Hyperintensities on Diffusion-Weighted Imaging Following Epileptic Seizures: A Case Report

**DOI:** 10.7759/cureus.73163

**Published:** 2024-11-06

**Authors:** Miharuka Yokosaki, Dai Agari, Yuta Maetani, Hiroki Ueno, Eiichi Nomura

**Affiliations:** 1 Department of Neurology, Hiroshima Citizens Hospital, Hiroshima, JPN

**Keywords:** diffusion-weighted imaging, drive camera, epileptic seizure, gyratory seizure, punctate hippocampus lesion, transient global amnesia

## Abstract

Punctate hippocampal hyperintensity (PHH) on diffusion-weighted imaging (DWI) is a well-known observation in patients with transient global amnesia (TGA), which is characterized by acute self-limiting episodes of anterograde and retrograde amnesia. These lesions occur unilaterally or bilaterally in the CA1 regions of the hippocampus, which are crucial for memory processes. PHH on DWI is well-documented in TGA but rare in other conditions. This case report describes a 56-year-old male bus driver who presented with PHH on DWI following epileptic seizures without TGA-like episodes. The patient experienced mild dizziness initially and subsequently lost consciousness while cleaning a bus. He was found collapsed outside the bus. The patient was slightly obtunded on arrival at the hospital and fully alert after admission. DWI showed bilateral PHHs in the CA1 regions. Recordings from multiple drive cameras installed on the bus clarified that he had developed gyratory seizures to the right, followed by focal onset bilateral tonic-clonic seizures. A seizure arising from the left hemisphere was suspected. As this was his first unprovoked seizure, the patient was closely followed up. This case demonstrates that PHH, a representative imaging feature of TGA, appeared with epileptic events but without TGA-like episodes. The occurrence of PHH in this context may broaden the clinical significance of PHH, with such occurrences helping to clarify the mechanisms underlying this condition.

## Introduction

Punctate hippocampal hyperintensity (PHH) is a well-established finding of diffusion-weighted imaging (DWI) in patients with transient global amnesia (TGA), which manifests as sudden onset of anterograde and retrograde amnesia and typically resolves within 24 hours [1]. PHH lesions are usually 1-5 mm in size and occur unilaterally or bilaterally in the CA1 region of the hippocampus, a critical area for memory processes [2]. PHH lesions generally become visible on DWI 24-72 hours after the onset of the TGA symptoms [3,4]. PHH is most commonly observed in TGA, but rare cases of PHH without TGA have been reported in patients with benign paroxysmal positional vertigo accompanied by vomiting, as well as headaches [5,6]. However, PHH has never been reported in patients with epileptic seizures without amnestic symptoms. This report describes a case of bilateral PHHs on DWI observed after epileptic seizures without associated TGA, which suggests that PHH can occur after epileptic seizures, thus expanding the understanding of the etiology of these lesions.

## Case presentation

**Figure 1:**
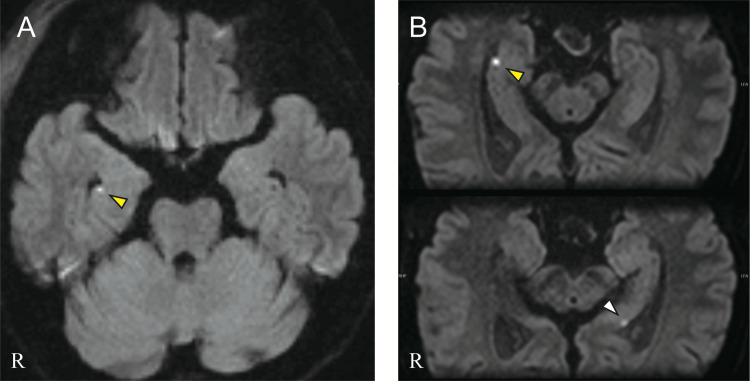
Diffusion-weighted imaging (DWI) showing punctate hippocampal lesions A: On the second day of hospitalization, DWI with 5 mm slices demonstrated a punctate hyperintensity in the right hippocampus (yellow arrowhead). B: On the third day, DWI performed parallel to the long axis of the hippocampus with 3 mm slices detected the previously observed punctate hyperintensity in the right hippocampus (yellow arrowhead) and a similar finding in the left hippocampus (white arrowhead). Both lesions were located in the CA1 regions.

A 56-year-old male bus driver presented to the emergency hospital because of consciousness loss. His medical history was unremarkable. The patient initially experienced mild dizziness while cleaning the inside of the bus. Subsequently, he lost consciousness after stepping outside. He was found collapsed outside the bus. On arrival at the hospital, the patient was slightly obtunded. Blood tests showed no signs of anemia and were otherwise unremarkable, although lactate levels were elevated at 3.1 mmol/L. Electrocardiography (ECG) and head computed tomography found no abnormalities. After admission, the patient was fully alert and had a clear recollection of the examination conducted on arrival. On the second day of hospitalization, electroencephalography revealed no interictal epileptiform discharges. DWI performed approximately 19 hours after symptom onset using 5-mm slices, demonstrated a punctate hyperintensity in the right hippocampus (Figure 1A) with low apparent diffusion coefficient (ADC) values. On the third day, follow-up magnetic resonance (MR) imaging with 3 mm slices detected the previously observed lesion in the right hippocampal CA1 region and a similar finding in the left hippocampal CA1 region (Figure 1B), with low ADC values. No abnormalities other than the hippocampal lesions were detected. MR imaging, including T1-weighted, T2-weighted, fluid-attenuated inversion recovery, and T2 sequences, did not reveal any abnormalities that could be considered potential causes of epilepsy. MR angiography showed no remarkable changes. Holter ECG monitoring, transthoracic echocardiography, and carotid artery ultrasonography did not reveal any findings indicative of a potential cause for the ischemic stroke.

Recordings from multiple drive cameras installed on the bus were obtained, which provided crucial insights into the patient's seizure semiology. These recordings revealed that the patient exhibited rightward gyratory movements, rotating four times, followed by figure-of-four posture with right arm extension, subsequently evolving into focal to bilateral tonic-clonic seizures (FBTCS). This seizure semiology strongly suggested a left hemispheric origin of the seizure, but this episode was the patient's first unprovoked seizure, and close follow-up was planned without initiating anti-seizure medications.

## Discussion

This case describes the transient loss of consciousness associated with bilateral PHHs on DWI. Initially, the cause of the consciousness loss was unclear due to the absence of eyewitness accounts. However, recordings from multiple drive cameras revealed that the patient had experienced a rightward gyratory seizure followed by FBTCS, suggesting a left hemispheric origin. Elevated lactic acid levels further supported a postictal state [7]. This case is noteworthy because PHH, typically seen in TGA, was observed in the context of epileptic events but without TGA-like episodes.

Whether the DWI hyperintensity lesions, particularly those associated with infarctions of the hippocampus, could have led to acute symptomatic seizures was crucial to establish. The lesions, which were evident on MR images the day after admission, may have occurred at the time of onset or later, as seen in diseases like TGA. However, the lesions were located in the CA1 area, similar to those observed in TGA. Hippocampal infarction typically presents with additional infarcts in regions supplied by the posterior cerebral artery [8]. Furthermore, the patient's symptoms did not align with the clinical features characteristic of hippocampal-onset mesial temporal lobe epilepsy, such as abdominal aura or automatisms associated with focal impaired awareness seizures [9]. Based on these observations, we believe that the hippocampal lesions in this case had appeared secondarily in association with the seizures rather than being immediate.

Gyratory seizures (GS) are characterized by rotation of the body axis by more than 180 degrees, with 360-degree or even greater rotations in the same direction occurring one or multiple times [10]. The basal ganglia may be involved in the manifestation of these epileptic seizures [11], but the mechanism remains unclear. A previous study of patients with refractory epilepsy who underwent comprehensive evaluation, including prolonged video-EEG monitoring, showed that GS is observed in both frontal and temporal lobe epilepsy [12]. Patients presenting with gyratory movements leading to a figure-of-four posture before evolving into FBTCS were all diagnosed with frontal lobe epilepsy. If the rotation direction corresponds to the initial head-turning, the seizure onset zone can be localized to the contralateral hemisphere. Therefore, the seizure semiology in the present case might similarly suggest that the seizure focus was located in the left frontal lobe.

In the present case, PHH was observed in the CA1 region of the hippocampus, which is consistent with the typical location of PHH seen in TGA [2]. One widely accepted hypothesis regarding the etiology of PHH associated with TGA posits that temporary blood flow disturbances occur in the hippocampal region, particularly in the CA1 area known for its vulnerability to hypoxia [3]. This localized ischemia may be triggered by various factors, including venous congestion, small arterial emboli, or cortical spreading depression [13-15]. Interestingly, infrequent reports of PHH occurring without TGA may suggest a complex relationship between the imaging findings and clinical presentation [16]. The Valsalva maneuver has been implicated as a potential trigger for venous congestion, leading to transient hypoperfusion in the hippocampal area. This mechanism has been proposed in the pathogenesis of PHH in patients both with and without TGA. In the present case of PHH associated with epileptic seizures, we hypothesize that the tonic phase of the seizure may have induced an increase in intrathoracic pressure, mimicking the effects of a Valsalva maneuver. This seizure-induced Valsalva-like effect could plausibly cause venous congestion, aligning with the proposed mechanism for PHH formation in TGA. This hypothesis might explain the initial occurrence of brain lesions on the right, contralateral to the presumed seizure onset side (on the left). More experience with similar cases may further elucidate the precise mechanisms underlying PHH formation across various neurological conditions, so clarifying its clinical implications, particularly in the context of epilepsy.

## Conclusions

PHHs were observed on DWI following an epileptic seizure in a patient without associated TGA-like episodes. The hippocampal lesions were located in the CA1 region, similar to those typically seen in TGA. The presence of PHH in the context of seizures suggests the need to consider mechanisms such as seizure-induced venous congestion, which could mimic the effects of the Valsalva maneuver, as one potential trigger for this condition. Further research is needed to better understand the mechanisms underlying PHH formation in various neurological conditions and its clinical implications in epilepsy.
